# Supplementary material for: Structure and function of rice hybrid genomes reveal genetic basis and optimal performance of heterosis
Source: Nat Genet. 2023 Sep 7;55(10):1745–56. doi: 10.1038/s41588-023-01495-8 (PMC10562254; doi:10.1038/s41588-023-01495-8)
Supplement: Supplementary file 1 — Supplementary Fig. 1. [file 41588_2023_1495_MOESM1_ESM.pdf]

nature genetics

Article

https://doi.org/10.1038/s41588-023-01495-8

Structure and function of rice hybrid genomes reveal genetic basis and optimal performance of heterosis

In the format provided by the authors and unedited

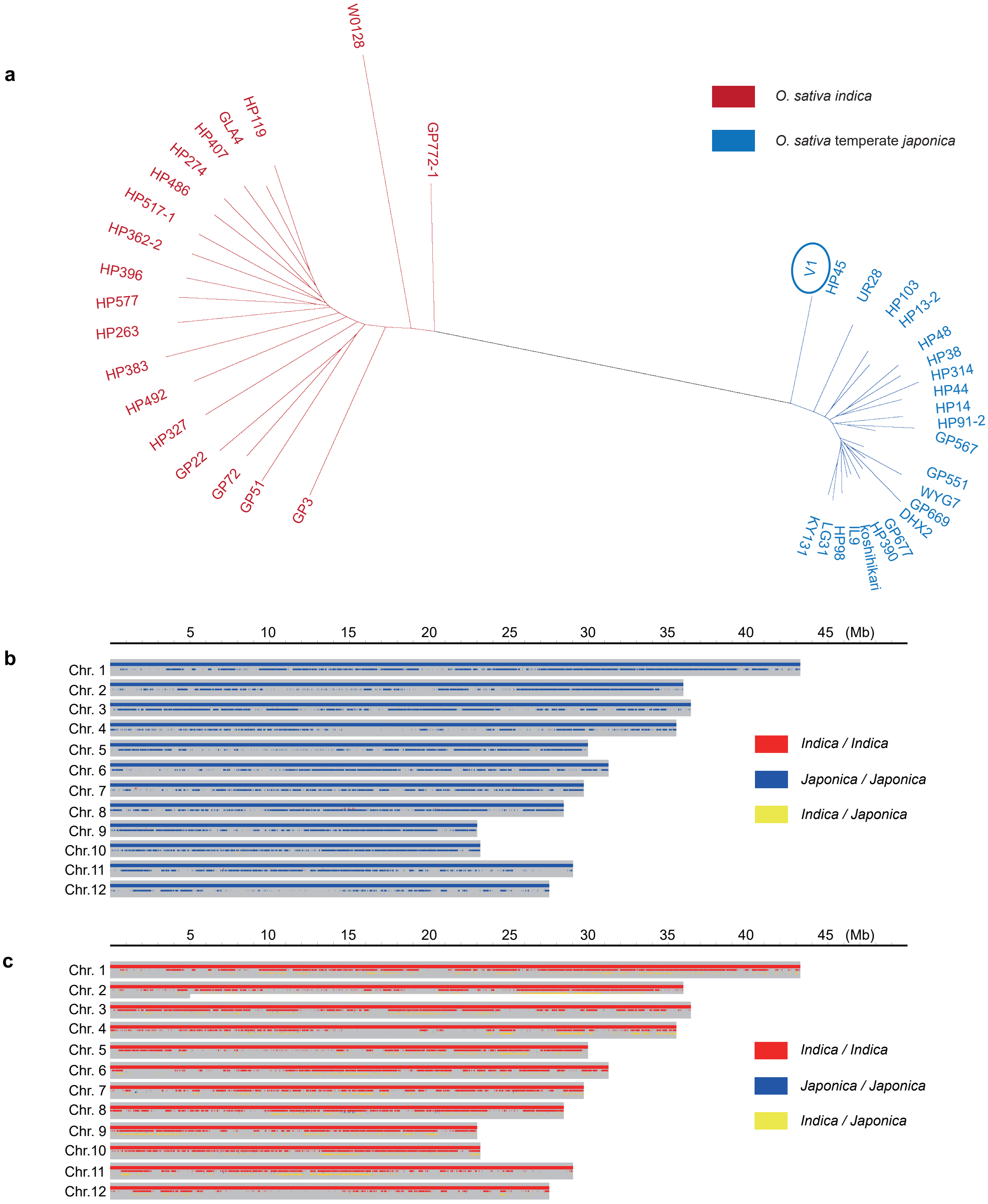

Supplementary Fig. 1 | Analyzing the population structure of samples to identify *indica-japonica* differentiated SNPs, and verifying their ability to differentiate *indica*- and *japonica*-origin sequences. a, Neighbor-joining tree of the 42 rice accessions used for *indica-japonica* differential SNPs identification. The tree was constructed using whole-genome polymorphisms. The accessions belonging to *O. sativa indica* and temperate *japonica* subgroups were respectively indicated by red and blue. **b,** The distribution of *indica*/*indica*, *indica*/*japonica* and *japonica*/*japonica* SNPs as well as segments across the whole genome of *japonica* cultivar Nipponbare. In the panel, the gray area represeted hypothetical chromosomes. In each gray shaded region, the lower stripe represented SNPs along the hypothetical chromosomal region, and the higher bar indicated the genomic sequence with its genotype judged by the SNP markers distributed in it. *Indica* SNPs and sequences were marked by red, *japonica* SNPs and sequences were in blue, and heterozygous SNPs and sequences were in yellow. In Nipponbare, the percentage of *indica* SNPs was 0.03%, *japonica* SNPs was 99.93% and heterozygous SNPs was 0.04%. And all the 199-SNP length fragments were judged as of *japonica*-origin by the procedure described in Methods. **c,** The distribution of *indica*/*indica*, *indica*/*japonica* and *japonica*/*japonica* SNPs as well as segments across the whole genome of *indica* cultivar Shuhui498. The percentage of *indica* SNPs was 95.39%, *japonica* SNPs was 0.03% and heterozygous SNPs was 4.58%. And all the 199-SNP length fragments were judged as of *indica*-origin.
