# Supplementary material for: Structure and function of rice hybrid genomes reveal genetic basis and optimal performance of heterosis
Source: Nat Genet. 2023 Sep 7;55(10):1745–56. doi: 10.1038/s41588-023-01495-8 (PMC10562254; doi:10.1038/s41588-023-01495-8)
Supplement: Supplementary file 2 — Reporting Summary [file 41588_2023_1495_MOESM2_ESM.pdf]

Corresponding author(s): Bin Han
Shihua Yang

Last updated by author(s): Jul. 18, 2023

Reporting Summary

Nature Portfolio wishes to improve the reproducibility of the work that we publish. This form provides structure for consistency and transparency in reporting. For further information on Nature Portfolio policies, see our Editorial Policies and the Editorial Policy Checklist.

Statistics

For all statistical analyses, confirm that the following items are present in the figure legend, table legend, main text, or Methods section.

n/a	Confirmed	
☐	☒	The exact sample size (*n*) for each experimental group/condition, given as a discrete number and unit of measurement
☐	☒	A statement on whether measurements were taken from distinct samples or whether the same sample was measured repeatedly
☐	☒	The statistical test(s) used AND whether they are one- or two-sided<br>*Only common tests should be described solely by name; describe more complex techniques in the Methods section.*
☐	☒	A description of all covariates tested
☐	☒	A description of any assumptions or corrections, such as tests of normality and adjustment for multiple comparisons
☐	☒	A full description of the statistical parameters including central tendency (e.g. means) or other basic estimates (e.g. regression coefficient) AND variation (e.g. standard deviation) or associated estimates of uncertainty (e.g. confidence intervals)
☐	☒	For null hypothesis testing, the test statistic (e.g. *F*, *t*, *r*) with confidence intervals, effect sizes, degrees of freedom and *P* value noted<br>*Give P values as exact values whenever suitable.*
☒	☐	For Bayesian analysis, information on the choice of priors and Markov chain Monte Carlo settings
☒	☐	For hierarchical and complex designs, identification of the appropriate level for tests and full reporting of outcomes
☐	☒	Estimates of effect sizes (e.g. Cohen's *d*, Pearson's *r*), indicating how they were calculated

Our web collection on statistics for biologists contains articles on many of the points above.

Software and code

Policy information about availability of computer code

Data collection

No software was used to collect data.

Data analysis

The details have been described in Method section.
1. Reads quality control was performed using Trimmomatic (version 0.38) with parameters 'ILLUMINACLIP:TruSeq3-PE.fa:2:30:10:2:true MAXINFO:50:0.6'. The clean reads were mapped against the rice genome IRGSP1.0 by BWA (version 0.7.1). Variation was detected by GATK (version 4.1.4.1).

2. Genome-wide nucleotide diversity was calculated using VCFtools (version 0.1.15) with 200kb sliding window. Kinship coefficient was calculated using EMMAX (version emmaxbeta-07Mar2010) and visualized using Cytoscape (version 3.8.2). Four-fold degenerate (4DTv) sites were identified by SnpEff (version 4.3t). PCA was performed by GCTA (version 1.93.2 beta). Ancestral components for hybrids were inferred by the ADMIXTURE program (version 1.3.0) and visualized using the R package pophelper (version 2.3.1)

3. GWAS was performed by the mixed linear model in the TASSEL software package (version 5.0 Standalone). The high-quality SNP data were further filtered by software PLINK (v.1.90b6.12 64-bit) to keep variants with a missing rate ≤10% and minor allele frequency ≥5%. Principal component analysis was performed using the input genetic markers by the software GCTA (version 1.93.2 beta), and the first two principal components were incorporated as the covariates to effectively account for population structure. Kinship matrix was generated based on the input genetic markers by "Kinship" function in TASSEL.
The dominance-effect/additive-effect (d/a) for association signal was calculated based on genotype effect estimated by TASSEL. The genotype effects of peak SNP in target association signal were chosen to calculate d/a index:
$a=|A-C|/2$
$d=M-(A+C)/2$

Furthermore, for QTLs mapping by F2 population, the index of d/a was estimated by IciMapping software (version 4.2.53).

4. The PVE by the candidate region surrounding the association signal was estimated according to previously report. A mixed linear model with multiple random effect was applied to estimate the variance components using the R package sommer (version 4.2.0.1).

5. Training a model based on the GBLUP method to predict breeding value. We used the GBLUP method in R package sommer to train the model.

6. Custom scripts and codes used in this study are provided in the GitHub repository (https://github.com/zlguu/Rice_Heterosis).

For manuscripts utilizing custom algorithms or software that are central to the research but not yet described in published literature, software must be made available to editors and reviewers. We strongly encourage code deposition in a community repository (e.g. GitHub). See the Nature Portfolio guidelines for submitting code & software for further information.

Data

Policy information about availability of data

All manuscripts must include a data availability statement. This statement should provide the following information, where applicable:

- Accession codes, unique identifiers, or web links for publicly available datasets
- A description of any restrictions on data availability
- For clinical datasets or third party data, please ensure that the statement adheres to our policy

Data availability statement is provided in Page 29, Lines 856-861:
All data supporting the findings reported here are available in the paper and supplementary files.
1. The raw DNA sequencing data of the 2,839 rice hybrid genomes used in this study are deposited in the NCBI Sequence Read Archive under study accession no. PRJEB53225.
2. Sample information and phenotype are provided as the Supplementary Information.
3. The publicly available website, incorporating resource applied in this study, is at http://ricehybridresource.cemps.ac.cn/#/.

Human research participants

Policy information about studies involving human research participants and Sex and Gender in Research.

Reporting on sex and gender	not involved
Population characteristics	not involved
Recruitment	not involved
Ethics oversight	not involved

Note that full information on the approval of the study protocol must also be provided in the manuscript.

Field-specific reporting

Please select the one below that is the best fit for your research. If you are not sure, read the appropriate sections before making your selection.

☒ Life sciences ☐ Behavioural & social sciences ☐ Ecological, evolutionary & environmental sciences

For a reference copy of the document with all sections, see nature.com/documents/nr-reporting-summary-flat.pdf

Life sciences study design

All studies must disclose on these points even when the disclosure is negative.

Sample size	All 2,839 rice hybrids are from the collections preserved at the China National Rice Research Institute in Hangzhou, China, including 1,495 hybrids rice accessions reported previously.
Data exclusions	No data was excluded from the phenotypic comparison analysis. Considering the TASSEL software was sensitive to outliers, the outliers was removed by “boxplot(data$phenotype, plot = FALSE)$out” script in R-4.1.0 for yield per plant, full grain number per plant, seed setting rate, valid panicle number and chalkiness in indica-indica hybrids, to better identify loci associated with phenotypic variation in indica-indica rice hybrids.
Replication	For morphological characteristics and yield components and relevant factors, they were investigated with three biological replicates for each sample. The heading date was recorded as the duration in days from the date of sowing to the emergence of first inflorescences above flag leaf sheath of five plants for each accession. With respect to grain quality-related traits, grains from mixed harvest were randomly selected to conduct investigation, with two replicates for each accession.

Randomization	We used all the samples to conduct phenotypic comparison, genomic structure analysis and GWAS (except for the outliers), and randomization was not involved. Furthermore, with respect to model construction, 6-fold cross-validation was used to estimate the accuracy of the model.
Blinding	The field work and phenotypic investigation of randomly-selected 67 pseudo-combinations out of all 1,102 combinations were parallel to the model construction and selection index calculation, and the analysis was under double-blind experimental control.

Reporting for specific materials, systems and methods

We require information from authors about some types of materials, experimental systems and methods used in many studies. Here, indicate whether each material, system or method listed is relevant to your study. If you are not sure if a list item applies to your research, read the appropriate section before selecting a response.

Materials & experimental systems

n/a	Involved in the study	
☒	☐	Antibodies
☒	☐	Eukaryotic cell lines
☒	☐	Palaeontology and archaeology
☒	☐	Animals and other organisms
☒	☐	Clinical data
☒	☐	Dual use research of concern

Methods

n/a	Involved in the study	
☒	☐	ChIP-seq
☒	☐	Flow cytometry
☒	☐	MRI-based neuroimaging
